# A Hypochondriacal Patient's Song

**Published:** 1888-09-15

**Authors:** 


### A HYPOCHONDRIACAL PATIENT'S SONG.

By a Hospital Nurse.

I.

Conceive me if you can,
A most acute young man ;
A pericarditis,
Chronic arthritis,
Double optic neuritis young man.

II.

A rose-coloured rash young man,
Disappearing on pressure young man ;
A convalescent typhoid,
Must solids avoid,
Though easily annoyed young man.

III.

A morbid and crabby young man,
A restless and flabby young man ;
A chronic œdema,
Perhaps pleurodynia,
Abdominal pains young man.

IV.

A dying of thirst young man,
A gluten and jambul young man ;
Potatoes and bread avoid,
Take saccharine tabloid,
Deceitful, diabetic young man.

V.

A fanciful, ailing young man,
A martyr to pain young man ;
A headachy, colicky,
Not at all frolicky,
Hysterical duffer young man.

VI.

A collapsed and last gasp young man,
Effervescing ammonia young man ;
Ultimate empyema,
And some emphysema,
Choking pleuritis young man.

VII.

A most obscure young man,
A getting worse young man ;
A not at all typical,
Most inexplicable,
Cleared up by post-mortem young man.

